# Watch your language: An exploration of the use of causal wording in veterinary observational research

**DOI:** 10.3389/fvets.2022.1004801

**Published:** 2022-10-24

**Authors:** Jan M. Sargeant, Annette M. O'Connor, Sarah C. Totton, Ellen R. Vriezen

**Affiliations:** ^1^Department of Population Medicine, Ontario Veterinary College, University of Guelph, Guelph, ON, Canada; ^2^Department of Large Animal Clinical Sciences, College of Veterinary Medicine, Michigan State University, East Lansing, MI, United States

**Keywords:** causal language, observational studies, veterinary, causation, epidemiology

## Abstract

Observational research may be conducted to predict an outcome or to identify associations between an intervention or risk factor (an “exposure”) and an outcome. However, the end goal of observational research often is to identify exposures that can be manipulated to improve an outcome, meaning that the aim is identify causal relationships. Causal inference from observational studies may be appropriate when an exposure-outcome of interest is identified, causal reasoning is used to identify confounders, confounders are adequately controlled, and theoretical issues, such as temporality, are considered. If these conditions are not met, causal inference cannot be made in an observational study. The objective of our study was to explore the use of causal language in veterinary observational studies, and to compare the use of causal language between studies that appear to be predictive or associational in purpose vs. those that appear to be exploring causal relationships. The dataset comprised 200 observational studies in veterinary species published between 2020 and 2022. The majority (117 out of 200) were cross-sectional studies. There were 48 studies that described an exposure-outcome of interest, and we considered these studies to be exploring potential causal relationships; of note, this liberal categorization would be anticipated to overestimate the proportion of studies suitably designed for causal inference. Overall, 172 studies (86%) used causal wording in at least one section of the article. Causal language was used in 128/152 (84%) of studies exploring predictions or associations; this language implies causation when it is not appropriate to do so. In studies designed such that causal inference might be possible, 44/48 (92%) used causal language in one or more sections. There were no substantive differences in the use of causal wording between observational study designs, exposure types, or whether the first author's affiliation was a country in which English is an official language. There is a need for authors of veterinary observational studies to explicitly state the purpose of the study (associational, predictive, or causal), and to use causal wording appropriately based on the aim of the study.

## Introduction

In research, the aim often is to determine whether an exposure (i.e., an intervention, or risk factor) affects or causes an outcome. When an exposure is associated with an outcome, there are three possible explanations for the association: chance (sampling error), bias or confounding resulting in non-associated factors appearing to be associated, or a causal effect (1). It is not possible to estimate the role of chance with certainty in an individual observational study. However, it is possible to minimize the risk of confounding by using randomization to assign study participants to exposure groups. Thus, randomized controlled trials (RCTs) provide the highest level of evidence for making inferences about causation. However, RCTs are criticized as having very selective populations, being conducted under ideal conditions, and having short follow-up times (2). Additionally, RCTs are not ethical or feasible for evaluating all possible exposures that may cause a specific health outcome. Therefore, observational study designs have an important role in causal health research.

Not all observational research is conducted to explore causality. There are three broad reasons why an investigator may conduct an observational study: purely to identify associations, to predict an outcome, or to identify potentially causal associations (wherein modifying the exposure variable would result in a more beneficial or less harmful outcome) (3). Suppose, for example, that an observational study is conducted in which the outcome is nasal cancers in long-nosed breed dogs. In a study where the aim is purely to identify associations, “exposure to second-hand smoke” may be one of a number of exposures for which data were collected, with the various exposures entered into a statistical model to identify those associated with the outcome. In this instance, the analysis would identify whether exposure to second-hand smoke was associated with the risk of nasal cancers. If the aim of the study was predictive, the investigators might include exposure to second-hand smoke in addition to other factors in a regression model with the intent of explaining as much of the variability in nasal cancer in long-nosed breed dogs as possible. The magnitude of the effect between risk factors and the outcome is not of interest in such a model, just the ability to predict the outcome. In the extreme, such a model could include the presence of ashtrays in the household as a variable in the model and inclusion of this variable may result in a strong predictive model. Finally, the driver of the research may be to know whether modifying exposure to second-hand smoke will reduce the risk of nasal cancers in long-nosed breed dogs. In this instance, the aim of the research is to make a causal inference. Causal inference may be defined as the evaluation of associations to estimate the causal effect of an exposure on an outcome in a population and is used to address questions about etiology (4).

A concern with observational studies is the potential for bias, in particular confounding bias, given that randomization to minimize differences in prognostic factors between exposure groups is not a design feature. If, however, the aim of the study is purely to identify associations or to predict the outcome, control of confounding is not necessary; confounding is a causal concept that does not apply to associational or prediction studies (3, 5). However, exchangeability between groups is a key hallmark of causal inference. Therein lies the conundrum; the researcher may be aiming to identify causal factors, and yet the researchers are using a study design that identifies associations, rather than “proving” a causal effect. Theoretical causal frameworks, such as Hill's criteria (6), which includes considerations of concepts such as temporality of exposure to outcome, strength of association, dose response, biological plausibility, and others, are in place to help decipher whether causality is likely for an association between an exposure and an outcome. From a study design perspective, many methods are available to appropriately control for confounding variables (7, 8).

How then do we approach causal inference in observational research? Ahern (5) argued that a 4-step roadmap for causal inference should be followed. Step 1 is to articulate that the question of interest is related to causal inference. Hernan (3) supports this by suggesting that being explicit about the causal objective removes ambiguity in the research question and avoids the need to use contrived wording to avoid the use of terms associated with cause. In practical terms, we argue that having a specific (*a priori*) exposure-outcome pair to evaluate in a study is a pre-requisite to meet this step. Step 2 in the proposed roadmap for causal inference is to “link the causal and statistical parameters through assessment of the assumptions under which they are equal (known as identifiability)” (5). Simplistically, this may correspond to the development of a Directed Acyclic Graph (DAG) or a causal pathway diagram. This step would identify a non-parametric biological relationship between potential confounding variables and the exposure-outcome pair of interest. Step 3 is to estimate the statistical parameters (e.g., the strength of association between the exposure and the outcome, including appropriate control of confounders). Step 4 is to interpret the findings, for example by a consideration of Hill's criteria (6).

Study design and type of exposure variable also may have implications for establishing causality. For instance, the cohort design begins with disease-free individuals who are followed over time to compare incident outcomes between exposure groups. Therefore, it is known that the exposure preceded the outcome, corresponding to Hill's temporality criteria and providing a stronger argument for causality than observational designs where temporality cannot be established with certainty. Similarly, if an exposure is time-varying, it can be challenging to determine whether the exposure preceded the onset of a disease outcome.

The wording used by an author can imply whether the relationship between an exposure and an outcome is, or is not, causal. Causal language refers to the use of words or phrases that state or imply that one entity influences another (9). In some instances, qualifying wording, such as “might” or “appears to” may be added to provide a weaker expression of causality (9). The use of language is important; readers of observational studies may not have the expertise to understand the caveats to causality in a manuscript and therefore, if causal wording is used, the reader may make a causal inference where it is not appropriate. Alternatively, deliberately avoiding the use of causal language when the aim is to identify potential means to impact an outcome may lead to contrived reporting and ambiguity (3, 10). The use of causal wording should be linked to the study purpose; if that purpose is associational or predictive, then the use of causal wording is not warranted. If, however, the purpose is causal inference, then causal wording may be appropriate in some sections of an article: for instance, when stating the objectives and methods, and in the discussion section. Previous research has explored the use of causal language in published papers in human healthcare (10, 11), obesity and nutrition (12), and social work (13). However, no such analysis has been conducted in veterinary observational research.

The aim of this study was to explore the use of causal language in veterinary observational studies in the context of whether the study aim appeared to be the identification of associations or prediction of an outcome, or whether the study had an underlying causal premise (liberally defined as stating an exposure: outcome pair of interest). Specifically, the objectives were to describe the use of causal language in objectives statements, titles and abstracts, and the full text of observational studies in veterinary species as well as to categorize the use of causal language based on the intended purpose of the study (association or prediction vs. causal inference). Additional objectives included comparison of the use of causal language by observational study design, and by type of independent variable (intervention, time-varying risk factor, or time-invariant risk factor). A post-hoc objective was to categorize the use of causal language based on whether the country affiliation of the first author corresponded to a country in which English is an official language.

## Methods

### Protocol

The methods for this study are described in a protocol that was developed prior to the conduct of the search and is available at https://osf.io/qk8hd.

### Eligibility

The data used for this study were obtained from published observational studies that met the following eligibility criteria:

- Analytical observational studies conducted in veterinary species or directly about veterinary species. For instance, a study evaluating risk factors for antibiotic use in cattle would be eligible, but a study evaluating veterinarian perspectives on antibiotic stewardship would not be eligible. Eligible veterinary species included dog, cats, equines, and common terrestrial food animals (dairy cattle, beef cattle, swine, meat or egg chickens, turkeys, sheep, and goats). Diagnostic test accuracy studies were not eligible.
- Published after January 1, 2020.
- Full text available in English.
- Available *via* University of Guelph library resources.

### Search strategy

The literature search was conducted in MEDLINE^®^ (*via* PubMed) on March 31, 2022, using the search string shown in Table 1 and based on information available in the title or abstract. The search was limited to articles published after January 1, 2020, but no language restrictions were included at the time of the search (language of publication was assessed during title/abstract and full-text screening). No gray literature search was conducted.

**Table 1:** Search to identify veterinary observational studies published after January 1, 2020 in Medline^®^ (*via* PubMed) conducted on March 31, 2022.

**String #**	**Search string (applied to terms in title/abstract)**	**# Hits**
1	“Veterinary” OR “bovine” OR “cattle” OR “dairy” OR “beef” OR “feedlot” OR “cow” OR “cows” OR “sheep” OR “lamb” OR “ovine” OR “goat” OR “goats” OR “caprine” OR “dog” OR “dogs” OR “canine” OR “cat” OR “cats” OR “feline” OR “swine” OR “hog” OR “hogs” OR “pig” OR “pigs” OR “porcine” OR “horse” OR “horses” OR “equine” OR “donkey” OR “donkeys” OR “poultry” OR “turkey” OR “turkeys” OR “broiler” OR “broilers” OR “hen” OR “hens” OR “layer” OR “layers” OR “chicken” or “chickens”	1,841,594
2	“Observational” OR “longitudinal” OR “case-control” OR “case control” OR “cohort” OR “cross-sectional” OR “risk factor” OR “risk factors”	2,071,545
3	(“2020/01/01”[Date—Publication]: “3,000”[Date—Publication])	3,531,699
4	“Overview” OR “review” OR “systematic review” OR “meta-analysis”	2,124,083
5	(#1 AND #2 AND #3) NOT #4	11,912

### Study selection

The search results were exported to DistillerSR^®^ (EvidencePartners, Ottawa, ON, Canada). Eligibility screening was performed in DistillerSR^®^ with screening questions corresponding to the eligibility criteria. Regardless of the study design label given, the assessment for eligibility was based on whether hypotheses were tested. This was done because study design labels may be inaccurate; in particular, it is common for studies labeled as case series to include a population-based cohort component (14), which would be an eligible study design. The form was pre-tested by two reviewers on 100 abstracts (JS and ST).

Following pre-testing, machine learning was used to assist in the selection of eligible studies. To create a training set for the internal artificial intelligence screening feature in DistillerSR^®^, two reviewers (JS and ST) independently screened the first 1,000 citations using information available in the title or abstract, with any eligibility conflicts resolved by consensus. Thereafter, the internal DistillerSR^®^ AI tool was used to select the remaining eligible citations based on information available in the title or abstract. Eligibility of the citations selected by the artificial selection tool in DistillerSR^®^ was confirmed by two reviewers working independently using the full text. Any disagreements were resolved by consensus. Citations were presented to the reviewers in a random order until the target of 200 eligible articles was obtained.

### Sample size considerations

No formal sample size calculation was conducted. Instead, the sample size was selected to allow meaningful percentages to be presented and was based on feasibility for the research group. Thus, the study was not powered to detect a specific magnitude of difference and, therefore, the results of hypothesis tests are not presented.

### Data extraction

All data extraction was conducted in DistillerSR^®^. Data were extracted from each article independently by two members of the research team, with any disagreements resolved by consensus or the input of the first author. The data extraction tool was pre-tested on 10 articles, with minor modifications to wording for clarity.

Information extracted at the study level included year of publication, country affiliation of the first author, species or commodity group (selecting all that applied from a list), type(s) of exposure(s) evaluated (time-varying, time-invariant, or intervention), and type of observational study design based on author description or, if not provided, based on description of study subject selection. An exception to assigning the study design based on author description was for studies described by the authors as case series that included a population cohort design component; these were designated as cohort studies for the purposes of the current study. Information also was extracted on whether any statistically significant findings were described in the abstract, whether there was one or more defined exposure: outcome pair(s) of interest (stated as an *a priori* decision, specifically mentioned in the title or objectives statement, or used to calculate the sample size), and whether the authors described the use of DAGs or causal pathway models to identify potential confounding variables. Whether the country affiliation of the first author represented a country in which English is an official language was categorized after data collection using internet searches of the country name to identify official languages.

Information was collected on the use of causal language and non-causal language in the objectives statement within the abstract, the title or abstract other than the objectives statement, the objectives statement in the main article, and anywhere in the body of the article other than the objectives statement. When causal wording was used, information was collected on whether the causal wording was always, sometimes, or never qualified and, if causal wording was qualified, what qualifying words were used. Checklists of non-causal, causal, and qualifying words were developed based on similar studies conducted in other disciplines (9, 13). Specific words or phrases from these checklists were selected only when used in the context of the hypotheses being examined. For instance, “correlated” would not be selected if the only use of that word was to describe the use of correlation coefficients. Similarly, the use of “association” would not be recorded as having been used if the only mention of the word was to state that “measures of association were ….”. Also, if authors were discussing the results of other studies using causal or non-causal wording, these words or phrases were not recorded.

### Data analysis

The results are presented descriptively, using absolute numbers. Data summarization was completed using Excel? Version 16.61.1 (Microsoft, 2022). The use of causal and non-causal language was categorized by whether the study was associational or predictive vs. whether the study could potentially evaluate causal inference (i.e., had a defined exposure-outcome of interest). In addition, the use of causal and non-causal language was categorized based on the study design, exposure variable type, and whether the first author's affiliation was from a country in which English is an official language.

### Protocol deviations

The distinction of whether wording was included in objectives statements vs. elsewhere in the article was not described in the protocol, but rather was added during the data collection form pre-test stage when it was noted that the use of causal language differed in some instances in the objectives statements vs. elsewhere. Although not described in the protocol, we added a comparison of causal language use based on whether the country affiliation of the first author represented a country where English is an official language. We also did not describe the categorization of language use between association or predictive studies and potential causal inference studies in the study protocol. However, we *post hoc* determined that this categorization would be the most informative approach for discussing the appropriateness of causal language in veterinary observational research.

## Results

Of the 1,000 initial titles and abstracts screened by two reviewers independently, 511 were deemed eligible. Using this sample, and to mimic the ~50% of citations that were deemed eligible, the DistillerSR^®^ AI tool was used with a cut-point for inclusion of 0.15, identifying a further 5,336 potentially relevant citations. These citations were randomly selected using an internal feature in DistillerSR^®^ for full-text screening until 200 relevant articles were identified. These formed the dataset for this study. A reference list for the included studies is available in Supplementary materials.

Descriptive characteristics of the included studies are shown in Table 2. The studies represented a range of veterinary species and types of exposure variables. The cross-sectional study design was the most common design, representing over half of the studies (117/200). Over 90% (187/200) of the studies had at least one statistically significant result described in the title or abstract. First author affiliations were from 51 countries. The 5 most represented countries were the UK (26 studies), USA (25 studies), Ethiopia (15 studies), Brazil (12 studies), and Italy (10 studies). English was an official language for the country of first author affiliation for 82/200 studies.

**Table 2:** Descriptive characteristics of 200 observational studies in veterinary species published between 2000 and March 31, 2022, used in an evaluation of the use of causal wording.

**Variable**	**Number of studies**
**Year of publication**	
2020	82
2021	82
2022	36
**Species or commodity group^a^**	
Canine	64
Feline	17
Equine	26
Poultry (chicken)	8
Bovine—dairy	39
Bovine—beef or veal	11
Bovine—use not specified	18
Porcine	10
Ovine	27
Caprine	19
**Types of exposure(s) evaluated^a^**	
Interventions	145
Time-varying risk factor	179
Time-invariant risk factor	157
**Observational study design**	
Cross-sectional	117
Case-control	20
Cohort	63
**Statistically significant findings described in title or abstract**	
Yes	187
No	13
**One or more defined exposure: outcome pairs of interest**	
Yes	48
No	152
**Presented a directed acyclic graph or causal diagram**	
Yes	5
No	195

a Multiple selections per article were possible; therefore, total number may sum to more than the total number of studies included (200).

An exposure: outcome pair of interest was defined in the objective or title in approximately one-quarter of the studies (48/200) (Table 2). However, only 5/200 studies included a DAG or a causal diagram. Only 1/200 studies included both a defined exposure: outcome pair of interest and a causal diagram. Therefore, we liberally defined a potential causal inference study as one in which an exposure: outcome pair was defined as the main interest of the study. Given that causal inference studies should have an exposure: outcome pair of interest, a causal diagram, and adequate control of confounding, our definition overestimated the number of studies in which causal inference might be appropriate.

Causal language was used in at least one section of the article for 172/200 studies (86%). Causal language was used in 128/152 (84%) of studies exploring predictions or associations; this language implies causation when it is not appropriate to do so. In studies designed such that causal inference might be possible, 44/48 (92%) used causal language in one or more sections. Table 3 shows the use of non-causal language, causal language, and whether causal language was always, sometimes, or never qualified, categorized for each article section, and categorized by whether the study appeared to be purely associational or predictive vs. potentially a causal inference study. Causal language commonly was used in all sections of the article, regardless of whether the study was potentially about causal inference.

**Table 3:** Use of causal and non-causal language in 200 observational studies in veterinary species published between 2,000 and March 31, 2022, based on whether the study purpose was purely to identify associations, predictive, or had a causal premise.

	**Number of studies**
**Wording in abstract objectives/hypotheses**	
**Used non-causal language**	
Association/predictive studies	112/152
Potential causal inference studies	35/48
**Used causal language**	
Association/predictive studies	9/152
Potential causal inference studies	7/48
**Qualified the causal language**	
Always qualified	1/16
Sometimes qualified	0/16
Never qualified	15/16
**Wording in abstract outside of objectives/hypotheses**	
**Used non-causal language**	
Association/predictive studies	147/152
Potential causal inference studies	47/48
**Used causal language**	
Association/predictive studies	54/152
Potential causal inference studies	25/48
**Qualified the causal language**	
Always qualified	5/79
Sometimes qualified	5/79
Never qualified	69/79
**Wording in objectives/hypotheses in main text**	
**Used non-causal language**	
Association/predictive studies	149/152
Potential causal inference studies	47/48
**Used causal language**	
Association/predictive studies	19/152
Potential causal inference studies	18/48
**Qualified the causal language**	
Always qualified	1/37
Sometimes qualified	1/37
Never qualified	35/37
**Wording in main text outside of objectives/hypotheses**	
**Used non-causal language**	
Association/predictive studies	152/152
Potential causal inference studies	48/48
**Used causal language**	
Association/predictive studies	122/152
Potential causal inference studies	43/48
**Qualified the causal language**	
Always qualified	9/165
Sometimes qualified	55/165
Never qualified	101/165

A wide variety of non-causal, causal, and causal qualifying words were used (Table 4). Common non-causal words across all sections of an article included association/associated, risk factor/sparing factor/protective factor, and higher/lower (or equivalent). The most commonly used causal words were increases/decreases, effects/affects/is effective, influences, and elevates/reduces/depresses. Common qualifiers included can/could (e.g., the exposure could influence the outcome) or may/might (e.g., the exposure might reducing the outcome).

**Table 4:** Description of non-causal, causal, and qualifying words using in different article sections.

	**Number of studies (out of 200) where a word or phrase was used**			
	**Abstract objectives/hypotheses**	**Title/abstract (other than objectives/hypotheses)**	**Objectives/hypotheses in main text**	**Main text other than objectives/** **hypotheses**
**Non-causal words**				
Association/associated	71	133	93	187
Risk factor/sparing factor/protective factor	96	117	105	146
Higher/lower/more or less likely/greater/lesser	1	114	9	180
Predicts/predictor/prognostic factor	15	30	17	72
Related to/relation(ship)	7	15	8	77
Correlated (with)		11	4	48
Explain(ed)/explanatory (variables)	–	2	–	49
Linked to	–	4	1	16
Varies with	1	5	–	11
**Causal words**				
Increases/decreases	2	50	13	110
Effects/affects/is effective	8	20	11	81
Influences	4	13	8	48
Elevates/reduces/depresses	–	14	4	37
Impacts	1	6	3	29
Contributes to	–	4	–	25
Cause(d)/consequence of	2	3	2	16
Is attributed to	–	1	–	10
Leads to	–	1	–	6
Improves/results in improvement	–	2	1	3
Is responsible for	–	1	–	4
Prevents	–	2	–	3
Results in	–	–	1	4
Proved to be	–	–	–	4
Altered by	–	–	1	3
Induces (an outcome)	–	–	–	4
Is successful	–	1	–	2
Enhances	–	–	–	3
Benefits from	–	–	–	2
Impairs	–	1	–	–
Driver of	–	1	–	–
Underlies	–	–	–	1
**Qualifying words**				
Can/could	–	6	–	25
May/might	1	1	2	23
Suggests that	–	2	–	11
Likely that	–	–	–	12
Appears to be	–	–	–	11
Possible that	–	–	–	3
Seems to	–	–	–	2
Presents some evidence	–	–	–	1
Thought to be	–	1	–	–

In Table 5 the use of causal wording by article section is summarized by type of observational study, whether the first author affiliation represented a country in which English is an official language, and by type of exposure variable. These results illustrate that causal language use is common across all study designs and exposure variable types and does not appear to be related to writing in English for non-English speakers (with the caveat that the official language of country of affiliation of the author may not reflect the native language of the author).

**Table 5:** Use of causal wording by study design, type of exposures examined, and whether the first author affiliation was from a country in which English is an official language.

	**Number of studies in which causal language was used**			
	**Abstract objectives/hypotheses**	**Title/abstract (other than objectives/hypotheses)**	**Objectives/hypotheses in main text**	**Main text other than objectives/** **hypotheses**
**Observational study design**				
Cross-sectional (*N* = 117)	5	37	11	89
Cohort (*N* = 63)	8	31	17	58
Case-control (*N* = 19)	3	11	9	17
Nested case control (*N* = 1)	0	0	0	1
**First author affiliation from a country with English as an official language**				
Yes (*N* = 82)	6	36	21	82
No (*N* = 118)	10	43	16	95
**Type of exposure variables**				
Intervention only (*N* = 3)	1	3	1	3
Time-varying only (*N* = 12)	1	4	3	7
Time-invariant only (*N* = 6)	0	2	1	3
Intervention and time-varying (*N* = 27)	7	14	7	25
Intervention and time—invariant (*N* = 11)	1	3	1	8
Time-varying and invariant (*N* = 37)	2	10	6	24
All three types (*N* = 104)	4	43	18	95

## Discussion

The results of this study illustrate that the use of causal language is common in observational studies and does not appear to differ substantially between study designs, exposure variable types, or based on whether authors are affiliated with an English-speaking country. Causal language was used in studies that did not include a defined exposure: outcome pair of interest (and thus could not be used for causal inference) as well as those studies that did. To the authors' knowledge, there has not previously been an evaluation of the use of causal language in veterinary observational studies. However, Cofield et al. (12) reported that causal language was used in 31% of observational studies of obesity and nutrition in humans, and its use appeared to be independent of funding source. Olarte Parra et al. (10) evaluated the use of causal wording in human medicine but restricted the evaluation to cohort and other longitudinal studies describing an outcome-exposure relationship. In their study, 48% of the 60 studies consistently used causal language, 20% inconsistently used causal language, and 32% consistently used non-causal language. Rubin and Parrish (13) evaluated the use of causal language in experimental and observational studies in social work research and reported that 60% of studies with designs that did not warrant conclusive causal inference used words or phrases that could be considered causal. Thus, our findings are in line with the results of previous research in disciplines other than veterinary science.

A premise of this study is that in observational study designs, causal inference, and therefore causal language, is inappropriate in some situations but may be appropriate in others. To make causal inference from an observational study, there should be a specific exposure: outcome pair of interest, confounding variables should be identified based on causal reasoning and adequately controlled in the design or analysis, and other issues such as temporality should be considered. If these conditions are not met, then the use of causal wording is inappropriate. In this study we used a very liberal definition of a potential causal inference study. For our classification, we did not require that an exposure: outcome pair of interest be explicitly defined as the primary comparison of interest (e.g., specifically designated or used to calculate the sample size); we merely required that one, or a small number of, exposure: outcome pairs were mentioned in the title or objectives statement. We did not require that a DAG or causal diagram be presented to explain the identification and rationale for selecting confounding variables, and we did not evaluate the methods used to control for confounding or the consideration of philosophical causal reasoning. Therefore, we would have misclassified studies as causal inference studies that should not have been, which would underestimate the inappropriate use of causal language. Because only 1 study included both steps 1 (explicit exposure: outcome pair) and 2 (causal diagram to identify confounding variables), it could be argued that this study was the only one that could potentially make a causal inference, although control of confounding and causal reasoning, such as Hill's guidelines, also would have to be assessed to determine if even this single study could make causal inference. However, it does raise the point that authors of observational studies should be explicit as to the overall aim of the study: identifying associations, predicting an outcome, or exploring possible causal relationships. Further, our approach to defining an exposure: outcome relationship might include associations that were identified after analysis, with manuscripts subsequently written to reflect an *a prior* hypothesis. Such issues are relevant to the validity of inference because they suggest random discovery of associations, but they are not germane to a discussion about causal language.

Based on our finding that only 48/200 of included studies included an exposure: outcome pair of interest, and that only 5 included a causal diagram, it appears that most of the observational research in veterinary medicine is intended to be predictive or associational. The reason for this is unknown. However, one might postulate that the large proportion of studies that are associational may be driven by data availability, rather than being question-driven research. Thus, it is important for authors of observational studies to be explicit about the purpose of their study. Purely associational research has value for generating hypotheses when little is known about a topic and predictive modeling can be useful for predicting the likelihood of an outcome, for instance for use in resource allocation or management decisions. If the study aim is to identify associations or to predict an outcome, it is not necessary to identify exposure: outcome pairs of interest, present DAGs, or control for confounding. It is interesting to note that many predictive or associational studies adjusted for covariates, often explicitly described as identification or control of confounding variables (data not shown), although no exposure of interest was identified. It is possible that this is related to lack of clarity by the authors as to the study purpose. However, causal language should not be used in these studies.

In contrast, if the purpose is to identify potentially causal relationships, then the criteria for doing so should be met, and authors need not shy away from using causal language when stating their objectives and in the results and discussion. In our study, ~8% of the studies that appeared to explore causal inference did not use causal language. A guidance for control of confounding and reporting of results in causal inference studies has been created by editors of respiratory, sleep, and critical care journal (4). These guidelines were developed by an *ad hoc* group of 47 editors representing 35 journals, and were intended to provide guidance to authors, peer reviewers, and researchers on the design and reporting of observational causal inference studies. The use of causal diagrams is advocated in this guidance, and the guidance also notes that models based on *p*-values or coefficient changes are not adequate for controlling for confounding. The potential use of this guidance for veterinary studies has not been evaluated. However, given the use of causal language in veterinary observational studies, there appears to be a need for discussion on the role of, and methods for, causal inference studies in veterinary medicine. This process could be led by researchers or journal editors and ideally would involve discussion and consensus among experts in the design and conduct of veterinary observational studies. There is precedent for consensus guidelines related to observational studies in veterinary science; the STROBE-Vet statement was developed by expert consensus and provides guidance for reporting veterinary observational studies (15, 16).

To use causal language appropriately, it is essential that study authors are aware of which words and phrases imply causation and which do not. To this end, the lists compiled for this study may serve as a resource for authors. The lists were derived from other studies, rather than by a consensus process. There may be value in collaboratively developing a list of causal and non-causal words that are agreed on, and recognized by, the veterinary observational research community.

A potential limitation of this study was to restrict inclusion to studies that could be accessed *via* University of Guelph library resources. However, no articles were excluded for this reason and so it was not a limitation. Another potential limitation of this study was the inclusion of only English-language studies. The decision to use only English-language studies was based on the language competency of the authors. Given the focus of this study on the use of language, our results may not be relevant for observational studies published in other languages. To some extent, we were able to explore this issue by comparing studies in which first author affiliations were from countries where English is an official language vs. other countries. However, as countries may have multiple official languages and country affiliation may not align with authors' mother tongues, misclassification of English-language competency may have occurred. It could be anticipated that misclassifying non-native English speakers as English speakers would tend to minimize differences for this categorization.

In summary, our results show that causal words and phrases are embedded in veterinary observational research, regardless the purpose of that research. Causal wording in observational studies is not appropriate unless there is a defined exposure: outcome pair of interest and adequate control of confounding variables, at a minimum. If these conditions are met, then authors should not avoid the use of causal wording. There is a need to define more explicitly the aim of a study, follow causal frameworks when the study aim is causal inference, and use causal wording when appropriate to avoid ambiguity.

## Data availability statement

The original contributions presented in the study are included in the article/Supplementary material, further inquiries can be directed to the corresponding author.

## Author contributions

JS, ST, and EV extracted data from published studies. JS drafted the manuscript. All authors contributed to the conceptualization of this work, read, and approved the final contents.

## Funding

No external funding was acquired for this project. Partial funding support was obtained from the University of Guelph Research Leadership Chair (Sargeant).

## Conflict of interest

The authors declare that the research was conducted in the absence of any commercial or financial relationships that could be construed as a potential conflict of interest.

## Publisher's note

All claims expressed in this article are solely those of the authors and do not necessarily represent those of their affiliated organizations, or those of the publisher, the editors and the reviewers. Any product that may be evaluated in this article, or claim that may be made by its manufacturer, is not guaranteed or endorsed by the publisher.
